# Supplementary material for: Identification of photoperiod-induced specific miRNAs in the adrenal glands of Sunite sheep (Ovis aries)
Source: Front Vet Sci. 2022 Jul 22;9:888207. doi: 10.3389/fvets.2022.888207 (PMC9354845; doi:10.3389/fvets.2022.888207)
Supplement: Supplementary file 1 [file Data_Sheet_1.ZIP › Supplementary file/miRDeep2 file.zip!/miRDeep2 file/Know_Structure/LP42A1/oar-mir-194.pdf]

miRBase precursor : oar-mir-194
Total read count : 4334
oar-miR-194 read count : 4330
remaining reads : 4

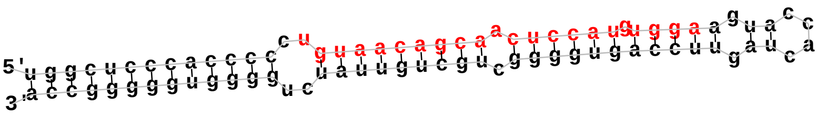

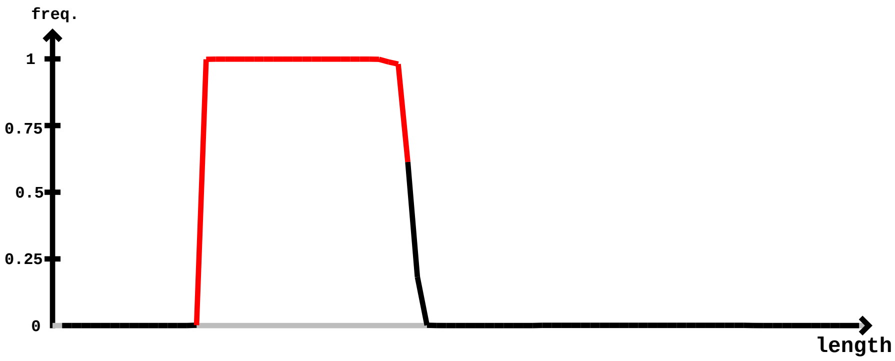


```
                 oar-miR-194
5'- uggcucccacccccuguaacagcaacuccaugugggaaguaccacuaguuccaguggggcugcuguuaucuggggugggggcca   -3'    exp
    (((((((((((((..(((((((((.((((((.(((((.((....)).))))))))))).)))))))))..)))))))))))))    reads      mm         sample
    .............cuguaacagcaacuccaugugg................................................    2          0          seq
    .............cuguaacagcaacuccauguggaa..............................................    4          0          seq
    ..............uguaacagcaacuccaug...................................................    3          0          seq
    ..............uguaacagcaacuccaugu..................................................    43         0          seq
    ..............uguaacagcaacuccaugug.................................................    34         0          seq
    ..............uguaacagcaacuccaugugg................................................    1590       0          seq
    ..............uguaacagcaacuccaugugga...............................................    1867       0          seq
    ..............uguaacagcaacuccauguggaC..............................................    16         1          seq
    ..............uguaacagcaacuccauguggaa..............................................    763        0          seq
    ..............uguaacagcaacuccauguggaag.............................................    4          0          seq
    ..............uguaacagcaacuccauguggaagu............................................    1          0          seq
    ...............guaacagcaacuccaugugga...............................................    1          0          seq
    ...............guaacagcaacuccauguggaa..............................................    2          0          seq
    .................................................ccaguggggcugcuguuaucug............    3          0          seq
    .................................................ccaguggggcugcuguuaucugg...........    1          0          seq
```
